# Role of nearshore benthic algae in the Lake Michigan silica cycle

**DOI:** 10.1371/journal.pone.0256838

**Published:** 2021-08-26

**Authors:** John A. Berges, Allison M. Driskill, Emily J. Guinn, Kaytee Pokrzywinski, Jessica Quinlan, Benjamin von Korff, Erica B. Young

**Affiliations:** 1 Department of Biological Sciences, University of Wisconsin-Milwaukee, Milwaukee, Wisconsin, United States of America; 2 School of Freshwater Sciences, University of Wisconsin-Milwaukee, Milwaukee, Wisconsin, United States of America; Mount Allison University, CANADA

## Abstract

Si cycling is linked with processes from global carbon sequestration to community composition and is especially important in aquatic ecosystems. Lake Michigan has seen dramatic fluctuations in dissolved silica (dSi) over several decades, which have been examined in the context of planktonic processes (diatom blooms), but the role of benthic organisms (macroalgae and their epiphytes) in Si cycling have not been explored. To assess significance of nearshore benthic algae in Si dynamics, we assembled dSi data from an offshore site sampled since the late 1980’s, and sampled off three Milwaukee beaches during 2005–19. Using colorimetric assays and alkaline digestion, we measured dSi, biogenic silica in particulate suspended material (pSi) and biogenic silica in benthic macroalgae (*Cladophora*) and epiphytic diatoms (bSi). Offshore, dSi increased about 1 μM per year from 25 μM in the late 1980’s to nearly 40 μM in 2019. Nearshore dSi fluctuated dramatically annually, from near zero to concentrations similar to offshore. Both *Cladophora* and its epiphytes contained significant bSi, reaching up to 30% of dry mass (300 mg Si g dry mass^-1^) of the assemblage in summer. Microscopic analyses including localization with a Si-specific-stain and X-ray microanalysis showed bSi in epiphytic diatom cells walls, but the nature and localization of Si in macroalgae remained unclear. A simple model was developed estimating Si demand of algae using the areal macroalgal biomass, growth rates inferred from P-content, and bSi content, and comparing Si demand with dSi available in the water column. This indicated that 7–70% of the dSi in water overlying nearshore benthic algal beds could be removed per day. Key elements of the Si cycle, including which organisms sequester bSi and how rapidly Si is recycled, remain unclear. This work has implications for coastal marine waters where large macroalgal biomass accumulates but bSi content is virtually unknown.

## Introduction

The global Si cycle profoundly affects the earth, from the draw-down of atmospheric CO_2_ due to chemical weathering of silicate minerals to driving changes in groups of silica-requiring organisms such as radiolarians, sponges and diatoms (one of the most productive groups of phytoplankton) over geological time [1]. In aquatic ecosystems, Si has been strongly linked to global carbon cycling, limitations of diatom primary production, and the efficiency of trophic transfer in freshwater lakes, streams and wetlands [2–4]. The short-term biogeochemical cycling of Si in aquatic ecosystems involves bioavailable dissolved silicate (dSi, usually considered as Si(OH)_4_ and SiO(OH)_3_^-^) and Si incorporated into organisms as biogenic silicate (bSi, hydrated polymeric silica). Modification of the biogeochemical cycle of Si by eutrophication is now clear in many aquatic ecosystems, but was first noted in the Laurentian Great Lakes [5].

There have been major changes in Si in the Laurentian Great Lakes, driven by anthropogenic nutrient inputs, invasive species and climate change [5–7]. Eutrophication from ~1954 to 1977 led to declines in dSi in the Laurentian Great Lakes and a build-up of bSi in sediments [6]. In the open waters of the lake, invasive Dreissenid mussels have depleted phytoplankton biomass and changed species composition [8], potentially changing water column dSi. In particular, declining spring diatom blooms have been associated with rising spring dSi concentrations [9–12]. At the same time, Dreissenid mussels have concentrated limiting phosphorus (P) in the nearshore benthos, described as a ‘nearshore shunt’ [13]. Mussel grazing removes water column particles, which both decreases light attenuation, and releases nutrients (especially P), for benthic filamentous algae (chiefly *Cladophora*), and dense epiphytic assemblages of silica-requiring diatoms [14, 15].

While changes to Si cycling in the open waters of the Great Lakes have been relatively well-appreciated, elements of benthic silica cycling have not been as well examined. Budgets for Si sources, sinks and regeneration in Lake Michigan are incomplete and based on the open lake [16] with little consideration for Si recycling in nearshore sediments [17], or the influence of Dreissenid mussels and benthic algal growth. To address this problem and assess the significance of benthic algal assemblages in nearshore silica dynamics, we measured water column dSi and bSi in algal assemblages at several nearshore sites over a 15-year period, and used a simple model to explore the potential Si demand of these assemblages in nearshore Lake Michigan. Our results demonstrate dynamic dSi in the nearshore, high bSi in benthic algae and the potential for benthic algae to have significant local effects on Si cycling in Lake Michigan and, by extension, other freshwater bodies.

## Materials and methods

### Field collections

In order to provide a baseline comparison for the nearshore, dSi data for the water column at the Fox Point station in the open waters of Lake Michigan (43.16 N, 87.67 W, 100 m depth) was assembled from seasonal data sets in the periods 1988–1991 and 2007–2009, which were described and published as averages by Engevold et al. [12]. In 2018 and 2019, additional dSi data was collected from Fox Point on vertical profiles collected from the RV *Neeskay*, essentially as previously described [12].

Three nearshore sites close to Milwaukee, Wisconsin (Atwater Beach 43.09 N, 87.87 W, Bradford Beach 43.06 N 87.87 W, and Linwood Beach 43.07 N 87.87 W) were sampled at intervals during February-December over several years between 2005 and 2019. In 2005, 2006, 2008 and 2018 sampling was conducted in the summer-fall period, in 2010 sampling focused on winter-spring, and in 2009 and 2019 sampling was conducted through all seasons. The sites are readily accessible, heavily influenced by riverine inputs, upwelling and nearshore beds of benthic algae. Samples were collected from surface water at 1 m depth by wading from shore and water used for dSi (filtrate) and suspended, particulate biogenic silicate (pSi, material captured on filters). In addition, detached and floating benthic algae at 1 m depth was collected for determination of biogenic Si (bSi). In 2006, attached benthic algae were sampled from the substratum by SCUBA diving at 10 m depth off all three beaches, using quadruplicate 20 x 20 cm quadrats. Areal benthic algal biomass (dry mass) was determined after samples were cleaned of invertebrates, sediment and stones, dried (65°C for 24 h) and weighed.

### Si analyses

Water samples were filtered (25 mm, 0.2 μm polycarbonate, Whatman Nucleopore), the filtrate used for dSi measurement [18] and filters with particulate material were stored frozen for later analysis of bSi. Benthic algal biomass was dried overnight at 65°C and both algal biomass and filters used for bSi analysis using high-temperature carbonate digestion (0.5% Na_2_CO_3_, 2 h extraction at 85°C, [19]). Carbonate digestion gave nearly identical results to hydroxide methods (using a shorter extraction at 100°C in 0.2 N NaOH, [20]), except that NaOH extracts of *Cladophora* samples were highly colored and bSi values more variable (cf. Krausse et al. [21], who noted the two methods compared favorably for freshwater diatoms). Particulate P content was measured on ashed algal biomass followed by acid digestion [15].

### Imaging of *Cladophora-*epiphyte assemblages

*Cladophora*-epiphyte assemblages were examined using light microscopy (Olympus BX-41) and scanning electron microscopy (SEM) in combination with elemental mapping (Hitachi S-4800 SEM with Bruker Quantax EDS system). In 2006 samples, Si incorporation into algal assemblages was examined by incubating algae for 2–4 days at 18 ^o^C, with ~30 μmol photons m^-2^ s^-1^ irradiance and 100 μM of the bSi incorporation label [2-(4-pyridyl)-5{[4-dimethylaminoethlamino-carbamoyl)-methoxy]phenyl}oxazole] (PDMPO, Lysosensor^TM^ Invitrogen, Carlsbad, CA) and samples examined using epifluorescence microscopy [22].

### Modeling

To assess potential significance of benthic algae in nearshore Si cycling, the Si demand represented by the biomass was estimated with a simple model based on data collected in 2005–6. First, we estimated benthic algal Si demand. The average Cladophora-epiphyte biomass (g m^-2^) and P content (mg P g dry mass^-1^) were measured from field samples at the three sites as described above. The *in situ* growth rate of benthic algal assemblages (d^-1^) was calculated using the measured P content of samples using a Droop-type relationship between growth rate and P content published for Lake Huron *Cladophora* by Auer and Canale ([23], Fig 7). We used average bSi contents determined from nearshore sampling to estimate algal Si content, as described above. Estimates of benthic algal areal coverage (m^2^) were derived from aerial photographs (from summer 2005, Source: SSEC RealEarth, UW-Madison, http://re.ssec.wisc.edu/?products=WICoast.100&center=43.086,-87.864&zoom=13), with nearshore area constrained by the 10 m depth contour, above which *Cladophora* growth is most abundant [24, 25]. The daily Si demand was calculated as the product of the measured average biomass and algal bSi content, the estimated daily growth rate, and areal coverage. Next, we calculated the dSi available in the water overlying the areas of each of the nearshore regions. The area (m^2^) was broken into depth contours 0–5 m and 5–10 m, by the mean depth in each area (i.e. 2.5 m or 7.5 m) and this was multiplied by the average dSi (10 μM) over the summer 2006 period. By dividing the dSi available by the daily Si demand, we calculated the potential proportion of dSi that could be used by the benthic algal assemblages and expressed this as a percentage.

### Statistical analyses

Data were analyzed using General Linear Model procedures, typically linear regression and Analysis of Variance, using logarithmic transformations when necessary to meet assumption of normality and homoscedasticity ([26], IBM SPSS Statistics version 26.0). Seasonal Mann-Kendall analyses [27] were used for offshore water-column dSi data, but uneven replication across season prevented this technique from being used for nearshore data.

## Results and discussion

### Rising offshore dSi over 20 years

**Fig 1:**
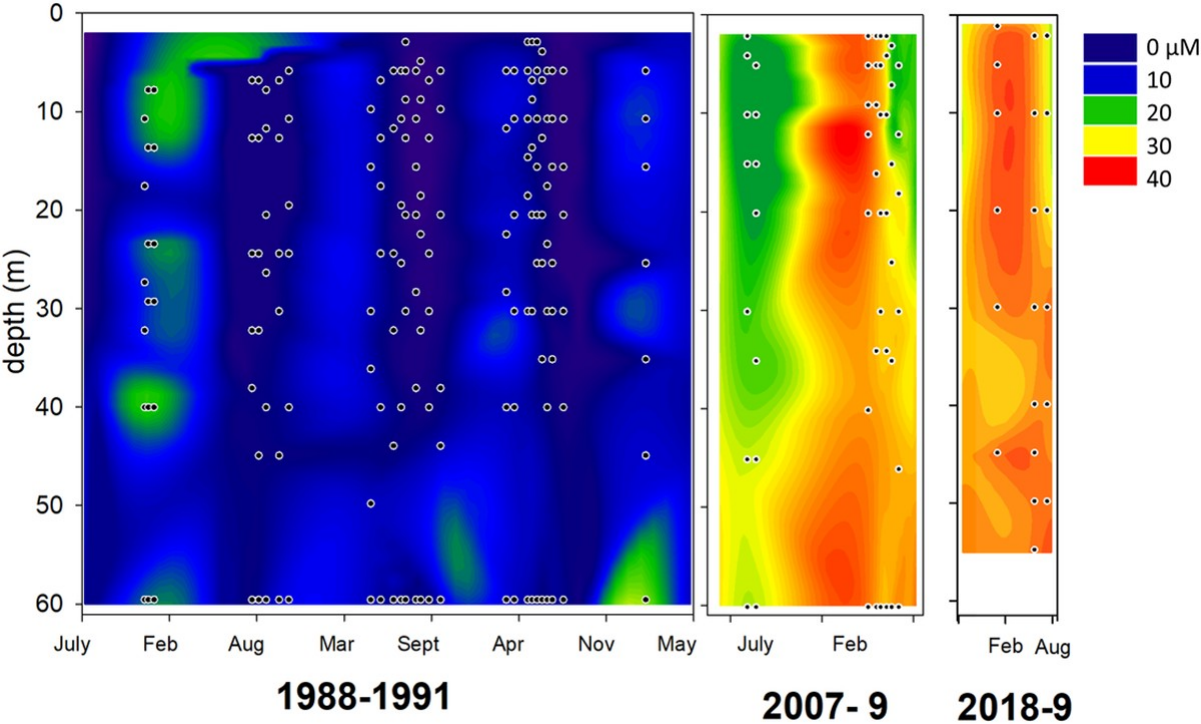
Dissolved silica concentrations (dSi) at Fox Point, Lake Michigan during the periods 1988–1991, 2007–2009 and 2018–9 through the top 60 m of the 100 m water column. Dots indicate sampling points.

Pelagic dSi in Lake Michigan has dramatically increased over recent decades. At the open water Lake Michigan station (Fox Point), dSi within the upper 60 m has increased from the period 1988–91, when typical dSi was on the order of 10 μM and maximum values were 20–25 μM, to during 2007–9 and 2018–9 when dSi averages close to 30 μM and values up to 40 μm have been recorded (Fig 1, [12]). A Seasonal Mann-Kendall analysis [27] demonstrated a highly significant increase in dSi over the period (P < 0.001), averaging about 1 μM per year (Sen’s slope 0.87 μM per year). Eutrophication from ~1954 to 1977 reportedly led to a 33 μM decline in dSi in the Laurentian Great Lakes and a build-up of sediment bSi [5]. But from a low of ~ 5 μM in 1988–90, the declining trend was reversed, a change attributed to phosphorus reduction efforts and invasive Dreissenid mussels which depleted phytoplankton biomass and changed phytoplankton species composition (especially decreased diatom abundance), resulting in declines in water column Si demand [8]. In Lake Michigan, declining spring diatom blooms have been associated with rising dSi in spring and a loss of seasonal variation in pelagic dSi. For example, at Fox Point Station Lake Michigan during 1986–1988 there were predictable seasonal dSi decreases from 20–25 μM in March to close to 0 in surface waters by July, while dSi remained at about 15 μM below the thermocline [12, 28]. Records from 2000 to 2006 show that surface dSi concentrations increased to close to those of deep water, and also that the seasonal oscillations in dSi were lost [11].

The observed changes in dSi are typically attributed to changes in diatom abundance, but such explanations are not completely satisfactory. Long-term dSi data from Environmental Protection Agency sampling between 1983 and 2008 do show dSi increases and slower seasonal draw-downs of dSi that can be attributed to reduced diatom blooms [10]. Finer scale sampling and primary production estimates provide evidence that the key changes in diatoms have occurred in the spring isothermal mixing period (April-May) when, until the late 1990’s, a diatom-dominated spring bloom occurred, but has subsequently been less dominant [9]. However, it might be expected that changes in spring diatoms blooms would be reflected in biomass or diatom species abundances later in the year, however few such differences are apparent during summer stratified period between the late 1980’s and the late 2000’s (see [12], S1 Fig). Moreover, declines in diatom blooms do not explain the water-column wide increases in dSi that have been observed. Missing from more recent analyses of the silica cycle has been consideration of pelagic or benthic bSi pools. Conley and Scavia [29] showed that at the 100 m deep Grand Haven Lake Michigan station during the April-May spring diatom bloom, a major pool (8–11 μM) of bSi developed in the >20 μm size fraction, which corresponded well with a draw-down in dSi from about 13 to 3 μM. The bloom rapidly died, fragmented and the bSi returned to dSi almost quantitatively [29].

Increasing dSi in deep water of Lake Michigan suggests additional changes, for example, in regeneration of bSi from sediments. Schelske et al. [30] noted that dSi remineralization depends heavily on the flux of material to the benthos and is very sensitive to regional differences in deposition; silica deposition has not been examined in Lake Michigan in some time [31] and an understanding of current diatom bloom dynamics at this level in Lake Michigan is lacking. However, very recent analysis of sediment cores taken at three stations in Lake Michigan found that bSi in cores (as a proportion of dry mass) was similar or increased from 1960 to 2009, while there was shift from larger to smaller-sized diatoms preserved in cores [32]. Increases in sediment bSi are difficult to reconcile with either declines in diatoms blooms or increased regeneration from sediments, and underline gaps in our understanding of the Lake Michigan silica cycle. Rising dSi represents a major biogeochemical shift in Lake Michigan, but such shifts are not unprecedented. Rising nitrate in Lake Superior is also occurring, hypothesized to be caused by alterations in nitrification [33].

### Nearshore Si dynamics

**Fig 2:**
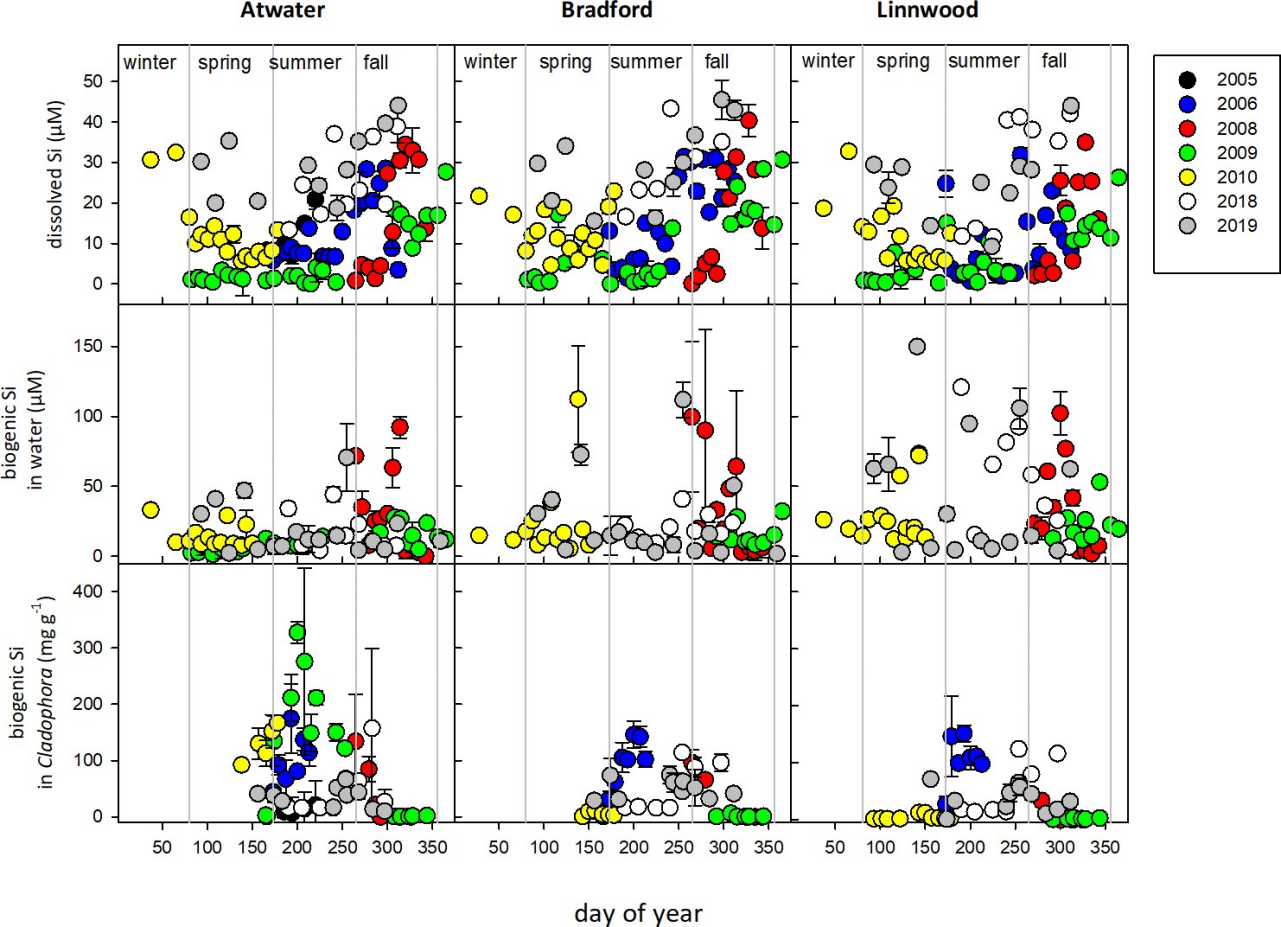
Dissolved silicate, suspended biogenic silica and biogenic silica in *Cladophora* assemblages in samples collected at 1 m depth at three Milwaukee-area beaches during 2005–2019.

In the western nearshore region of Lake Michigan, there were significant seasonal variations in Si pools. dSi varied widely, fluctuating between deep-lake values of over 30 μM to less than 1 μM within a space of days, in some years (Fig 2). Nearshore dSi fluctuations were examined by clustering values into seasons (spring, summer, fall and winter) and analyzing using a three-way ANOVA with location, year and season as factors. There was a significant interaction between year and season (P < 0.05), thus data were compared by year using Tukey post-hoc comparisons. No significant differences were found among the three locations (Atwater, Bradford and Linnwood, P > 0.05), however, while in 2005–2011, dSi in spring and summer were significantly lower than in fall and winter (P < 0.05), no significant seasonal differences were detected in 2018 or 2019 (P > 0.05). Particulate biogenic Si suspended in nearshore water (pSi) examined using a three way ANOVA (location, season, year) showed no significant differences (P > 0.05), though highest values tended to occur in fall and winter (Fig 2). The bSi content in macroalgal biomass collected from the three locations ranged from a few mg Si (g dry mass)^-1^ in June when macroalgal biomass is just starting to appear in the water, to over 300 mg Si (g dry mass) ^-1^ (i.e. over 30% of dry mass) in late July—August at Atwater Beach (Fig 2). Analysis by three-way ANOVA (location, year, season), showed significant interactions between location and year, and season and year (P < 0.001 in both cases), requiring separate Tukey post-hoc comparisons by year and location. *Cladophora* bSi values were significantly higher in 2005 and 2006 than in other years (P < 0.05). In the years 2005–2011, bSi values were higher in summer than other seasons (P < 0.05), but not significantly different among winter, spring and fall, and also significantly higher at the Atwater site versus the Linwood or Bradford Beach sites (P < 0.05). These differences observed in bSi among seasons and sites in 2005–11, did not hold in 2018 and 2019 (P > 0.05 in both cases). Although we have no objective measures of changing *Cladophora* growth or appearance on beaches over the course of this study, subjectively, beach accumulation was considerably worse in the earlier years, and this is reflected in declining frequency of Google searches by the public for information on “Cladophora” over the period (S2 Fig), suggesting that the algal biomass may have been more obvious to the public in the earlier period. In contrast, Kuczynski et al. [34], were able to use closures of a Lake Ontario beach as an index of *Cladophora* blooms because a County Health Department had defined “excessive Cladophora on the beach” as a criterion. It is also worth noting that Lake Michigan water levels have fairly consistently increased over the same time period (S2 Fig).

Increases in nearshore dSi are most likely driven by upwelling from deep lake waters; the frequency and magnitude appear to be consistent with patterns of measured and modelled upwelling [35]. River inputs are an alternative source of dSi, but US Geological Survey records for the common inflow of the Milwaukee, Menomonee and Kinnickinnic Rivers to Lake Michigan (Station 4087000, waterdata.usgs.gov) show modest average dSi concentrations of 245.1 (± 143.1) μM over the period 1980 to 2009, with no clear evidence of temporal trends, and no correlation between river discharge volume and dSi measured at nearshore sites. However, we have not adequately assessed bSi in river inputs because such measurements have rarely been taken. Riverine bSi is dominantly from terrestrial vegetation, and there is evidence that this plays a role in global silica cycling equivalent to that of oceanic diatoms [36]; river bSi as a component of the “terrestrial silica pump” also needs to be considered.

### Si in benthic algal biomass

**Fig 3:**
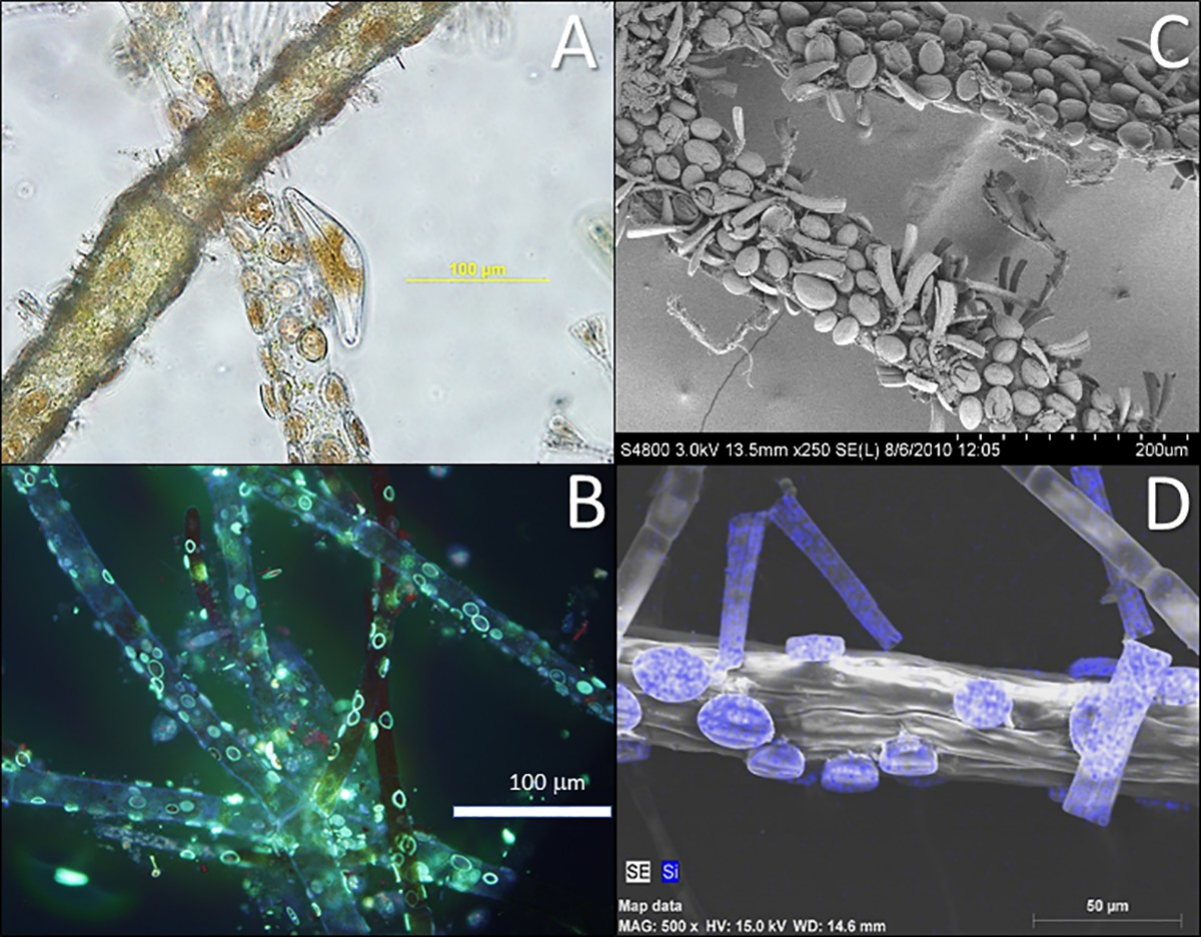
**A**. Light microscopy image of *Cladophora* filament with encrusting diatom epiphytes. **B**. Epifluorescence microscopy image of PDMPO-labeling of bSi (pale green) accumulation in epiphytic diatoms on surfaces of *Cladophora* filaments. **C**. Scanning electron microscope (SEM) images of *Cladophora* filaments with typical dense late-summer diatom epiphyte load. **D**. SEM elemental mapping of Si (blue dots) on *Cladophora* and epiphytes—elemental spot analysis over *Cocconeis* diatom cells yielded signals for C ~0.6 cps(eV) and Si >7.5 cps(eV) compared to *Cladophora*, C ~0.6 cps(eV), Si <0.1 cps(eV). Scale bars are as marked.

*Cladophora* typically showed dense epiphyte loads, dominated by diatoms (Fig 3). Species composition was similar to that described by Young et al. [15]: the most common species were *Cocconeis* sp., *Gomphonema* sp., *Tabellaria flocculosa*, *Rhoicosphenia curvata*, and *Cymbella* sp. along with *Dinobryon* sp., with filamentous cyanobacteria (including *Fischerella* sp. and *Pleurocladia lacustris*) present (Fig 3A and 3C). Elemental mapping over SEM surfaces showed highest Si localization within these epiphytic diatoms (Fig 3D), and epifluorescence microscopy of samples labelled with PDMPO showed Si incorporation in epiphytic diatoms, but only red chlorophyll fluorescence in *Cladophora* filaments (Fig 3B). PDMPO staining mechanism is not entirely clear but the basis of this oxazole dyes (originally developed for studying intracellular pH) is strongly pH-dependent and depends strongly on surface chemistry and concentration [37], so labelling of algal bSi maybe challenging in multicellular algae like *Cladophora*. Nevertheless, it seems likely that significant bSi in benthic algal assemblages is contributed by the epiphytic diatoms. However, in 2005, we were able to obtain relatively epiphyte-free “green” *Cladophora*. In July samples, *Cladophora* with typical epiphyte loads averaged 166 ± 68.4 mg Si (g dry mass)^-1^, while *Cladophora* with low epiphyte load had just 9.61 ± 1.79 mg Si (g dry mass)^-1^. Malkin et al. [14] published the only other comparable data set on benthic algal Si from a 2 m deep station in Lake Ontario in 2005 from spring through autumn, using hot NaOH extraction for bSi. While water column dSi was much lower (2.7–8.3 μM), bSi for the *Cladophora*-epiphyte assemblages was similar to the present study: 56–224 mg Si (g dry mass)^-1^, they also reported cultured *Cladophora* (without epiphytes) had only 4 mg Si (g dry mass)^-1^. Combining our summer estimates of bSi in the *Cladophora*-epiphyte assemblage, our P content measured in benthic samples in 2006 (see above), and average C and N contents determined on *Cladophora* samples collected in 2005–6, we can approximate the molar C:N:P:Si stoichiometry of the assemblage as 331:21:1:62. Previous Lake Michigan Si budgets have not included these benthic algal assemblages [38] and they clearly could represent significant local Si pools, though their impact on the overall lake budget is more limited because of the relatively small area of the nearshore region relative to the open waters of the lake.

*Cladophora* itself clearly contains some bSi, though its structural or physiological roles are unclear. Moore and Traquair [39] showed that growth of *Cladophora* was promoted by Si and inhibited by the silicate analog GeO_2_ and they speculated that electron dense areas in *Cladophora* cell walls might be sites of Si deposition. It is not especially surprising that *Cladophora* might contain bSi because it in the evolutionary lineage leading to higher plants and many angiosperms, including freshwater macrophytes, take up dSi [2]; *Elodea*, *Potamogeton* and *Myriophylum* species use bSi structurally and contain 2–70 mg Si (g dry mass)^-1^ ([40–42], Jack, Young and Berges unpublished). Freshwater phytoplankton groups contain bSi including some chrysophytes and even certain chlorophytes [43], but Si in diverse groups has not been incorporated into budgets or models; the current Lake Michigan Eutrophication Model (LM3-Eutro) only associates dSi uptake with planktonic diatoms [44]. Marine cyanobacteria in the genus *Synechococcus* from the Eastern equatorial Pacific Ocean accumulated Si and exhibited Si:P ratios approaching that of diatoms, and in fact the water column inventory of Si associated with cyanobacteria exceeded that in diatoms [45]. Potential Si pools in freshwater cyanobacteria, which are increasing in abundance in the Great Lakes [11], is unknown. In marine macroalgae, Markham and Hagmeier [46] showed negative growth effects of GeO_2_ in several species, suggesting requirements for Si uptake, bSi deposition has been associated with wound healing in *Saccharina japonica* kelp sporophytes [47], and a red seaweed showed evidence of increased temperature and irradiance stress when Si was less that 50 μM [48]. There is clearly a need to re-evaluate the pools and role of silica in broader taxonomic groups within aquatic ecosystems.

### Modelling of nearshore Si demand

We used some simple calculations to contextualize nearshore Si cycling by benthic algal biomass. Using data collected in 2005–6, we calculated dSi demand of the *Cladophora*-epiphyte assemblages in nearshore waters off the three beach sites, using a combination of aerial photos (S3 Fig) for areal coverage, *in situ* sampling for areal biomass, and growth rates derived from an established relationship with internal P-content [23]. Areal benthic algal bSi were calculated by multiplying biomass by bSi content to yield 43.7–279 mmol m^-2^ across the three sites (Table 1). These values are quite comparable to areal benthic algal bSi in Lake Ontario at a 2 m deep station: 22.7 mmol m^-2^ in spring, rising to 490 mmol m^-2^ at peak *Cladophora* biomass in summer [14]. P content of *Cladophora*-epiphyte assemblages ranged from 0.6 to 3 mg P g dry mass^-1^, very comparable to those found in Lake Huron [23], and mean values for samples from the three nearshore sites resulted in growth rates estimates between 0.4 to 0.6 d^-1^, based on Auer and Canale’s [23] data which found nutrient-replete net growth rates of 0.714 d^-1^. Determining the overlying water volume from depth intervals, and assuming an average 10 μM dissolved silicate (based on summer 2005–2006 values, Fig 2), we calculated that Si demand by the benthic *Cladophora*-epiphyte assemblages could account for 7 to 70% of dSi in the overlying water each day (Table 1). This substantial proportion of available dSi would easily explain the observed nearshore fluctuations in dSi (Fig 2) during the growing season. These calculations represent a theoretical maximum, and *Cladophora* cannot effectively access the whole overlying water column to deplete dSi, and *Cladophora* growth is unlikely to be maintained at consistently high rates and in step with epiphyte growth for many weeks. Nonetheless, the fluctuations in dSi in nearshore waters are demonstrated over several years of sampling, and the potential effects of the *Cladophora*-epiphyte assemblage on Si demand and cycling are evident.

**Table 1:** Modelled effects of the *Cladophora*-epiphyte assemblages on dSi at three Lake Michigan nearshore sites.

Site	μ (d^-1^)	Cover (x 10^6^ m^2^)	Biomass (g m^-2^)	bSi Content (mg g^-1^)	Si demand (tonnes d^-1^)^a^	Available Si (tonnes)^b^	% Si used (d^-1^)
Atwater	0.48	3.10	70.3	111.2	11.6	16.4	71.0
Bradford	0.58	3.14	10.8	109.9	2.15	9.45	22.7
Linnwood	0.58	1.75	10.8	113.7	1.26	16.7	7.5

Growth rates (μ) were estimated from *Cladophora* P-content, cover determined using aerial photographs, biomass from measurements of samples collected *in situ*, and bSi content determined in samples collected from the nearshore. The % Si used (d^-1^) represents the maximum daily Si demand of benthic *Cladophora*-epiphyte assemblages relative to that available in the overlying water column.

^a^Based on modeled growth rate (from P content), biomass and bSi content

^b^Based on an average dSi of 10 μM and volume of overlying water.

Importantly, we currently have very little idea where the bSi that is taken up by the *Cladophora*-epiphyte assemblage is stored, how labile it is, or where it goes after the assemblages die and break down. Because *Cladophora* coverage and biomass can be extensive in the nearshore (exceeding 80% of the benthic surface area and reaching up to 260 g dry mass m^-2^, [49]), the need to understand links to P cycling and model its growth dynamics have been appreciated [50]. However, while there is evidence of P-limitation in *Cladophora* (e.g. the presence of alkaline phosphatase activity [15], nutrient stoichiometry [25]), benthic algae in Lake Michigan also shows evidence of secondary limitation by dSi at concentrations below approximately 14 μM; nutrient enrichment with P + N+ Si show greater effects than enrichment without Si [51]. Furthermore, if we consider the benthic diatoms in the assemblage, there is evidence that they may be capable of extraordinary dSi uptake and uptake kinetics may not show saturation at typical lake dSi [52]. The critical importance of attached algae (including diatoms) in lake and river ecosystems with respect to food web effects has been recognized [53], but the roles of these groups in biogeochemical nutrient cycling are also significant and need further consideration.

### Critical gaps in understanding

In both the open lake and nearshore, recycling of bSi is a particularly critical component of Si budgets, yet our understanding is dated and fragmented. In open Lake Michigan waters, 80–100% of bSi may be recycled annually [5, 54, 55] and just 5% of bSi due to diatom production was estimated to be buried annually [56]. Schelske [38] completed a mass-balance and found that Lake Michigan contrasted sharply with Lake Superior in that Si demand by diatoms after the winter dSi maximum was 71% in Michigan vs. only 8% in Superior, a difference attributed to the eutrophication and P additions to Michigan. Nearshore Lake Michigan has received less attention than the open lake, but although we have now demonstrated seasonal depletion of dSi (Fig 2) we still know little about nearshore bSi recycling. In another deep lake system, Lake Malawi, riverine inputs were more significant as ~25% of Si input to the epilimnion (mostly as bSi within phytoliths from maize and grasses), but 75% Si still came from vertical exchange of Si-rich water from depth, and only 7 to 11% of diatom production becomes permanently buried [57]. In the shallower Lough Neagh, Northern Ireland, seasonal recycling of Si in the sediments is the major source of dSi to planktonic diatoms and benthic invertebrates play a major role in remineralization [58]. Indeed, Quigley and Vanderploeg [59] demonstrated the effectiveness of the benthic amphipod *Diporeia* in digesting diatom frustules, but it is also worth noting that *Diporeia* has significantly declined in Lake Michigan following the invasion by Dressenid mussels [32, 60]. Alternatively, the activity of invertebrates such as amphipods may also suppress dSi regeneration from sediments by burying bSi deposits [17]. There is little doubt that the invasion of Dreissenid mussels into Lake Michigan and other lakes has radically changed food web dynamics, and the cycling of phosphorus [13]. Since the examination of Si in Lake Michigan in the 1980’s [56], invasion and expansion of mussels and establishment of benthic algal blooms have quite likely altered Si cycling and pools, and a re-evaluation of these is needed. There is no doubt that changes in silica recycling can have profound effects on aquatic ecosystem function. In eutrophic Lake Kasumigaura (Japan), a three-decade-long increase in dSi, driven by sediment release and resuspension, has resulting in increases in diatoms and decreases in cyanobacteria in the phytoplankton [61].

## Conclusions

In conclusion, we have shown that benthic macroalgae and their epiphytes constitute a significant pool of bSi in nearshore Lake Michigan with potentially significant effects on dSi, but many key elements of the nearshore silica cycle such as rates of recycling of algal bSi pools remain poorly understood. Our work also has implications for coastal marine waters where large biomasses of macroalgae accumulate but bSi pools within this biomass, or contribution to Si cycling is virtually unknown.

## Supporting information

S1 Fig Comparison of diatom abundances at offshore Lake Michigan sites between the late 1980’s and 2008. Average counts of diatoms in samples collected June through August at two Lake Michigan stations at 100 m depth. 1985–8 data from Sandgren and Lehman (Sandgren CD, Lehman JT. Response of chlorophyll a, phytoplankton and microzooplankton to invasion of Lake Michigan by Bythotrephes. Verh. Int. Ver. Theor. Angew. Limnol. 1991; 24:386–92), 2008 data from Simmons et al. (Simmons LJ, Sandgren CD, Berges JA. Problems and pitfalls in using HPLC pigment analysis to distinguish Lake Michigan phytoplankton taxa. J. Great Lakes Res. 2016; 42: 397–404).
(PDF)

S2 Fig Comparison of popularity of Google searches for “Cladophora” with mean Lake Michigan water levels. Relative popularity of Google searches for the term “Cladophora” in Wisconsin, 2004 through 2019 by month, assessed with Google Trends (trends.google.com). Mean water level in Lake Michigan from US Army Corp of Engineers (www.lre.usace.army.mil/Missions/Great-Lakes-Information/Great-Lakes-Information-2/Water-Level-Data/).
(PDF)

S3 Fig Images showing the nearshore regions of three Milwaukee areas beaches examined in *Cladophora* modeling. Aerial photographs (Source: SSEC RealEarth, UW-Madison, re.ssec.wisc.edu/?products=WICoast.100&center=43.086,-87.864&zoom=13) and matching geo-referenced charts with depth contours (created by the authors in ESRI’s ArcGIS v.10.6; no copyrighted material was used) for three sites near Milwaukee, Wisconsin. Regions outlined in red represent the extent of benthic *Cladophora* distributions selected down to the 10 m depth contour.
(PDF)
