# Supplementary material for: Role of nearshore benthic algae in the Lake Michigan silica cycle
Source: PLoS One. 2021 Aug 26;16(8):e0256838. doi: 10.1371/journal.pone.0256838 (PMC8389419; doi:10.1371/journal.pone.0256838)
Supplement: S1 Fig — Average counts of diatoms in samples collected June through August at two Lake Michigan stations at 100 m depth. 1985–8 data from Sandgren and Lehman (Sandgren CD, Lehman JT. Response of chlorophyll a, phytoplankton and microzooplankton to invasion of Lake Michigan by Bythotrephes. Verh. Int. Ver. Theor. Angew. Limnol. 1991; 24:386–92), 2008 data from Simmons et al. (Simmons LJ, Sandgren CD, Berges JA. Problems and pitfalls in using HPLC pigment analysis to distinguish Lake Michigan phytoplankton taxa. J. Great Lakes Res. 2016; 42: 397–404). (PDF) [file pone.0256838.s001.pdf]

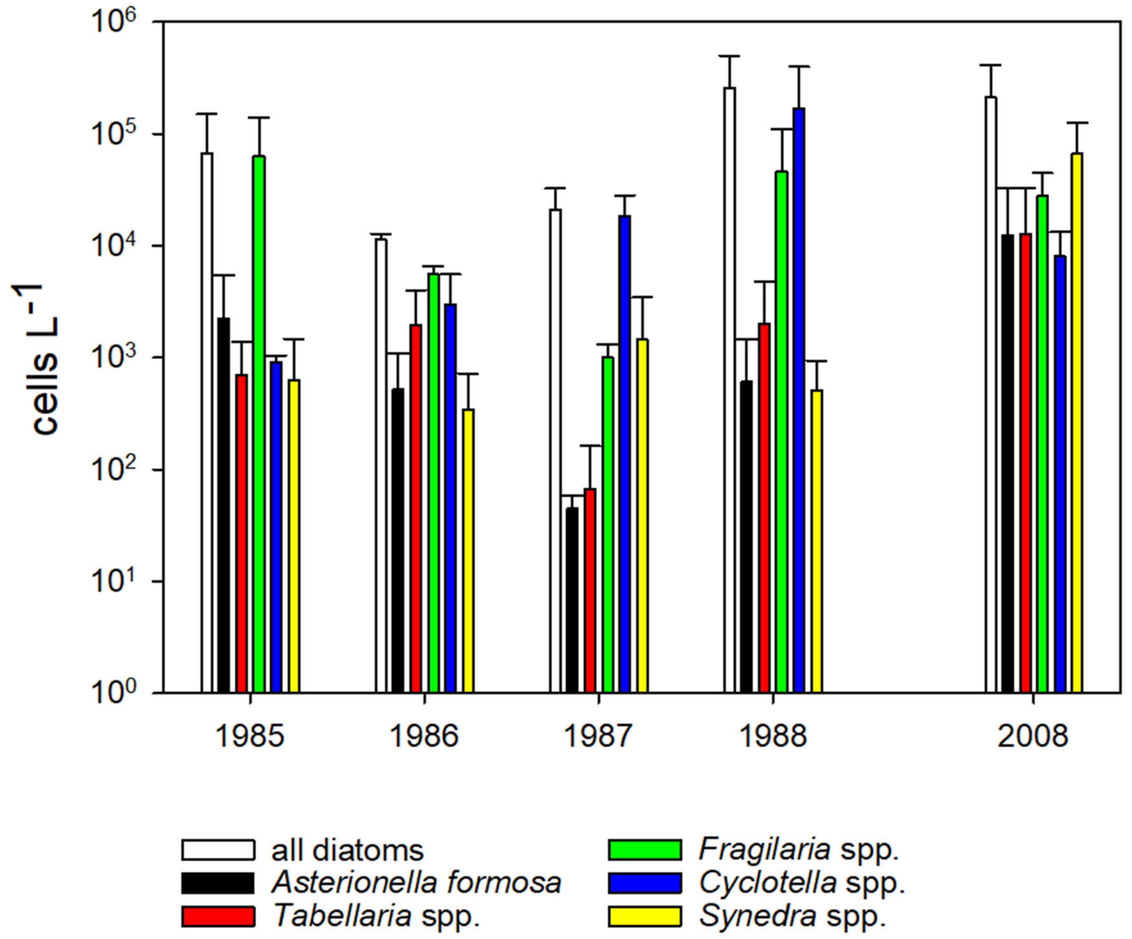

S1 Fig. Comparison of diatom abundances at offshore Lake Michigan sites between the late 1980's and 2008. Average counts of diatoms in samples collected June through August at two Lake Michigan stations at 100 m depth. 1985-8 data from Sandgren and Lehman (Sandgren CD, Lehman JT. Response of chlorophyll *a*, phytoplankton and microzooplankton to invasion of Lake Michigan by *Bythotrephes*. Verh. Int. Ver. Theor. Angew. Limnol. 1991; 24:386-92), 2008 data from Simmons et al. (Simmons LJ, Sandgren CD, Berges JA. Problems and pitfalls in using HPLC pigment analysis to distinguish Lake Michigan phytoplankton taxa. J. Great Lakes Res. 2016; 42: 397-404).
