# Supplementary material for: Role of nearshore benthic algae in the Lake Michigan silica cycle
Source: PLoS One. 2021 Aug 26;16(8):e0256838. doi: 10.1371/journal.pone.0256838 (PMC8389419; doi:10.1371/journal.pone.0256838)
Supplement: S2 Fig — Relative popularity of Google searches for the term “Cladophora” in Wisconsin, 2004 through 2019 by month, assessed with Google Trends (trends.google.com). Mean water level in Lake Michigan from US Army Corp of Engineers (www.lre.usace.army.mil/Missions/Great-Lakes-Information/Great-Lakes-Information-2/Water-Level-Data/). (PDF) [file pone.0256838.s002.pdf]

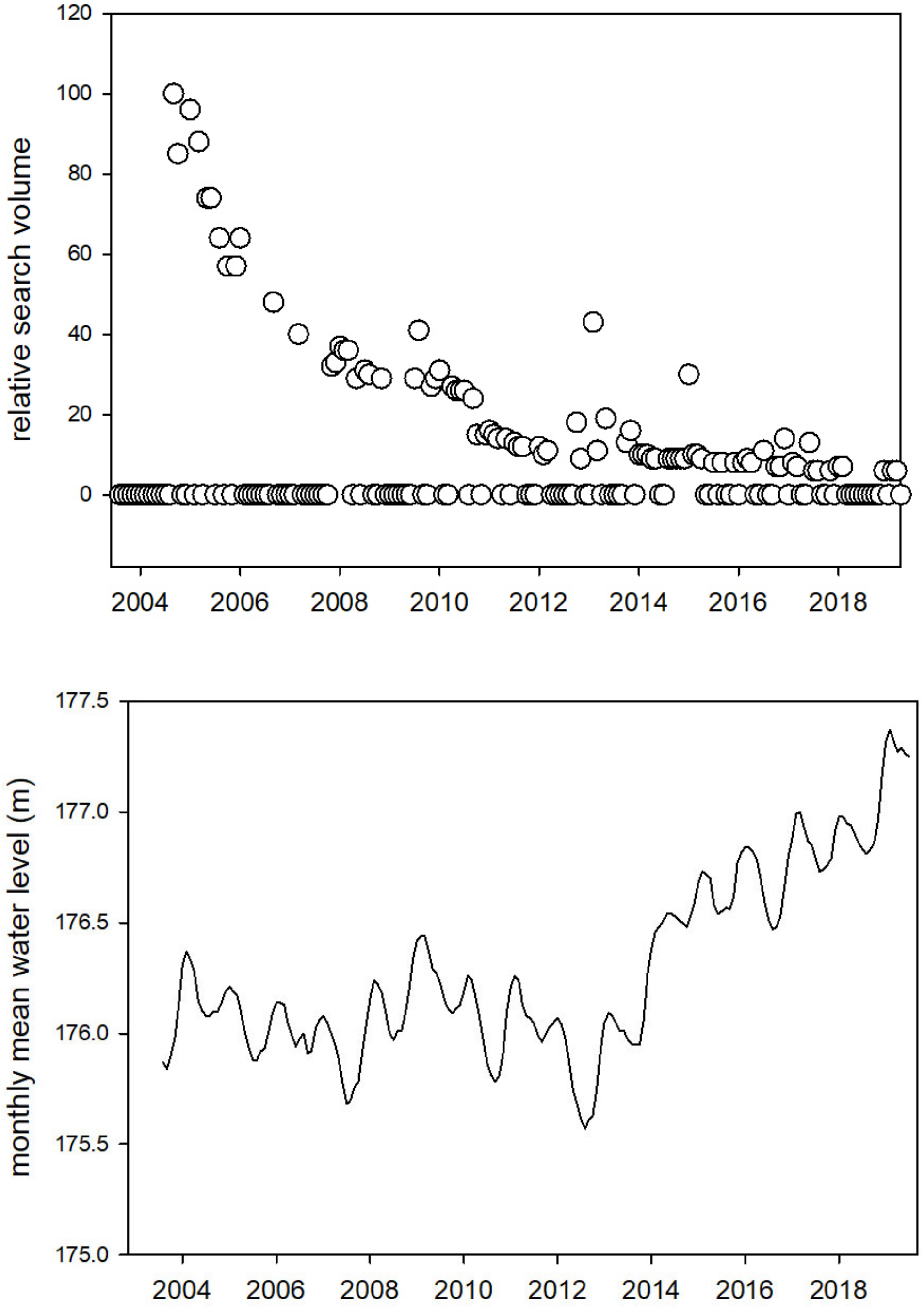

S2 Fig. Comparison of popularity of Google searches for "Cladophora" with mean Lake Michigan water levels. Relative popularity of Google searches for the term "Cladophora" in Wisconsin, 2004 through 2019 by month, assessed with Google Trends (trends.google.com). Mean water level in Lake Michigan from US Army Corp of Engineers (www.lre.usace.army.mil/Missions/Great-Lakes-Information/Great-Lakes-Information-2/Water-Level-Data/).
