# Supplementary material for: Role of nearshore benthic algae in the Lake Michigan silica cycle
Source: PLoS One. 2021 Aug 26;16(8):e0256838. doi: 10.1371/journal.pone.0256838 (PMC8389419; doi:10.1371/journal.pone.0256838)
Supplement: S3 Fig — Aerial photographs (Source: SSEC RealEarth, UW-Madison, re.ssec.wisc.edu/?products=WICoast.100¢er=43.086,-87.864&zoom=13) and matching geo-referenced charts with depth contours (created by the authors in ESRI’s ArcGIS v.10.6; no copyrighted material was used) for three sites near Milwaukee, Wisconsin. Regions outlined in red represent the extent of benthic Cladophora distributions selected down to the 10 m depth contour. (PDF) [file pone.0256838.s003.pdf]

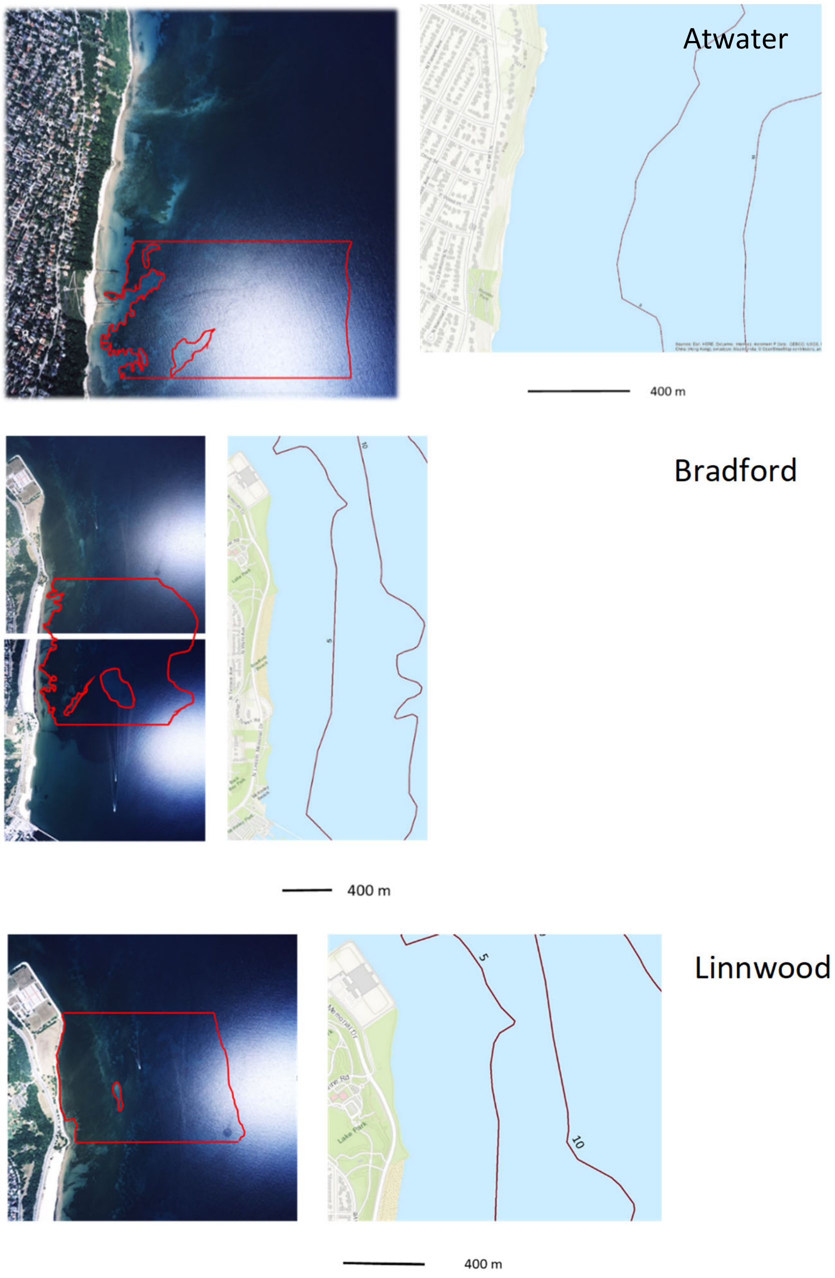

S3 Fig. Images showing the nearshore regions of three Milwaukee areas beaches examined in *Cladophora* modeling. Aerial photographs (Source: SSEC RealEarth, UW-Madison, re.ssec.wisc.edu/?products=WICoast.100&center=43.086,-87.864&zoom=13) and matching geo-referenced charts with depth contours (created by the authors in ESRI's ArcGIS v.10.6; no copyrighted material was used) for three sites near Milwaukee, Wisconsin. Regions outlined in red represent the extent of benthic *Cladophora* distributions selected down to the 10 m depth contour.
